# The genus *Fleischmannia* in Argentina, Bolivia, Brazil and Paraguay (Eupatorieae, Asteraceae)

**DOI:** 10.3897/phytokeys.57.5784

**Published:** 2015-12-02

**Authors:** Harold Robinson

**Affiliations:** 1 Department of Botany, MRC 166, National Museum of Natural History, P.O. Box 37012, Smithsonian Institution, Washington, DC. 20013-7012

**Keywords:** *Fleischmannia*, Argentina, Bolivia, Brazil, Paraguay, new taxa

## Abstract

Species of the genus *Fleischmannia* from Argentina, Bolivia, Brazil and Paraguay are reviewed, and keys are provided that cover the species in each country. New taxa described are *Fleischmannia hatschbachii*, *Fleischmannia matogrosensis*, Fleischmannia microstemon var. paniculata from Brazil, *Fleischmannia hassleri* from Paraguay and *Fleischmannia neei* and *Fleischmannia steinbachii* from Bolivia, and one new combination for a *Fleischmannia prasiifolia* variety is provided. The additions bring the total known species of the genus to 102.

## Introduction

*Fleischmannia* is a genus of annual to perennial, often scrambling, herbs in the tribe Eupatorieae concentrated in the Central America and the northern and central Andes. One species occurs in eastern North America and a few occur in eastern Brazil. Prior to the present study the genus contained 97 known species. Recent efforts to clarify the species of have concentrated on Mesoamerica (Robinson 2015), and northern and western South America (Robinson 2001, 2008). In these areas, the delimitation of species has proven more complicated than initially expected. Meanwhile, the study of the genus from farther south in South America has been subjected to the lowest form of taxonomy, a process that I refer to as identification work. Some individual specimens have been put aside over the years and are now subjected to new studies in more detail. The species have again proven to be more complicated than expected. Even countries with few species such as Brazil and Paraguay have presented some difficulties, and Bolivia has many names in use without clear distinctions and many more recent collections that do not match any of the described species.

## Materials and methods

Specimens used in the study were those accumulated over the last 150 years in the U.S. National Herbarium. Studies were restricted to use of the dissecting and compound microscopes.

## Results

The species of *Fleischmannia* in southern and eastern South America are reviewed here by country starting with Argentina, Paraguay and then Brazil, based on material accumulated over the years at the U.S. National Herbarium (US). Treated last is Bolivia with its 12 species. In the process, new species are described and keys are provided.

### Argentina

Only three species are credited to the country, none of them endemic. The three can be distinguished by the following key:

**Table:** 

1	Erect plants with ascending branches spreading at less than 45° angles; heads in large corymbiform clusters	***Fleischmannia prasiifolia***
–	Scrambling or leaning plants with branches spreading at 45–90° angles; inflorescence lax with long branches bearing small clusters of heads	**2**
2	Pappus bristles ca. 30, mostly not contiguous; Andean plants	***Fleischmannia schickendantzii***
–	Pappus bristles ca. 40, contiguous; plants of Paraná River system	***Fleischmannia dissolvens***

For the subscandent *Fleischmannia dissolvens* (Baker) R.M. King & H. Rob. of northeastern Argentina, see the treatment of the Paraguay species, and for the more erect or sprawling Andean species, *Fleischmannia prasiifolia* (Griseb.) R.M. King & H. Rob. and *Fleischmannia schickendantzii* (Hieron.) R.M. King & H. Rob., see the treatment of the Bolivian species.

### Paraguay

Two species have been collected in Paraguay. One previously only reported from Argentina and the other undescribed. The specimens of both species have previously been mostly mistakenly identified as *Fleischmannia prasiifolia* (Griseb.) R.M. King & H. Rob., a species now known not to be from Paraguay.

**Table:** 

1	Plants subscandent or scrambling; branches spreading at 60–90° angles; leaf blades membranaceous, mostly glabrous between the veins, without glandular dots abaxially	***Fleischmannia dissolvens***
–	Plants erect or leaning; branches ascending, spreading at less than 45° angles; leaf blades herbaceous, densely pubescent on both surfaces, with small glandular dots abaxially	***Fleischmannia hassleri***

#### Fleischmannia dissolvens

Taxon classificationPlantaeAsteralesAsteraceae

(Baker) R.M. King & H Rob., Phytologia 19: 203. 1970.

Eupatorium dissolvens Baker, Fl. bras. 6(2): 308. 1876. Type: Argentina. Entre Rios, Parana, *Christie 59* (holotype K).

##### Description.

Scrambling perennial herb ca. 1 m tall, with branches widely spreading at 45–90° angles; stems pale to dark brown, terete, striated, hispid to stiffly puberulous. Leaves opposite; petioles slender, 0.5–2.0 cm long; leaf blades triangular, membraneous, mostly 3.0–5.5 cm long, 2.0–3.5 cm wide, widest near basal 5^th^, base obtusely cuneate, margins with 5–10 sharp teeth, apex narrowly acute to slightly acuminate, surfaces puberulous on larger veins, sparsely pilosulous to subglabrous between veins, without glandular dots or hairs; triplinervate from petiole, with main veins brownish. Inflorescence a lax panicle with elongate branches, heads in cymiform clusters at tips of elongate branches; most nodes with foliiform bracts 1.5–2.0 cm long; branches and peduncles densely puberulous, peduncles 3–13 mm long. Heads ca. 7 mm high, 5 mm wide; involucral bracts ca. 30 in 3–4 series, basal involucral bracts narrowly ovate to lanceolate, 2–3 mm long, puberulous outside, inner bracts narrowly oblong, with short-acute tips. Florets ca. 17 in a head; corollas violet. ca. 2.5 mm long, basal tube ca. 0.5 mm long, throat ca. 2.2 mm long, lobes ca. 0.6 mm long, narrowly triangular, with numerous monoseriate hairs outside; anther thecae ca. 0.9 mm long, apical appendage ca. 0.2 mm long; style branches slender. Achenes 2.2–2.7 mm long, slightly and narrowly paler on ribs, ribs setuliferous; pappus whitish, of ca. 40 slender contiguous bristles ca. 3 mm long.

##### Specimens seen.

Paraguay: Guaira: Cordillera de Ybytyruzú, Cerro Acaté, secondary vegetation on base of mountain along southern side, 25°55'S, 56°15'W, 700 m, herb 1 m, flower pink, 17 Feb. 1989, *E. Zardini & R. Velásquez 11100* (FCQ, MO, US); Guaira: Cordillera de Ybytyruzú, road to Polilla, Cerro Acatí, forest, 25°55'S, 56°10'W, 800 m, shrub 1 m, 23 July 1989, *E. Zardini & R. Velásquez 13604* (FCQ, MO, US).

The species has previously been reported only from Entre Rios in Argentina. The Paraguay localities are farther up river.

#### Fleischmannia hassleri

Taxon classificationPlantaeAsteralesAsteraceae

H. Rob. sp. nov.

urn:lsid:ipni.org:names:77151273-1

##### Type.

**Figure 1.**
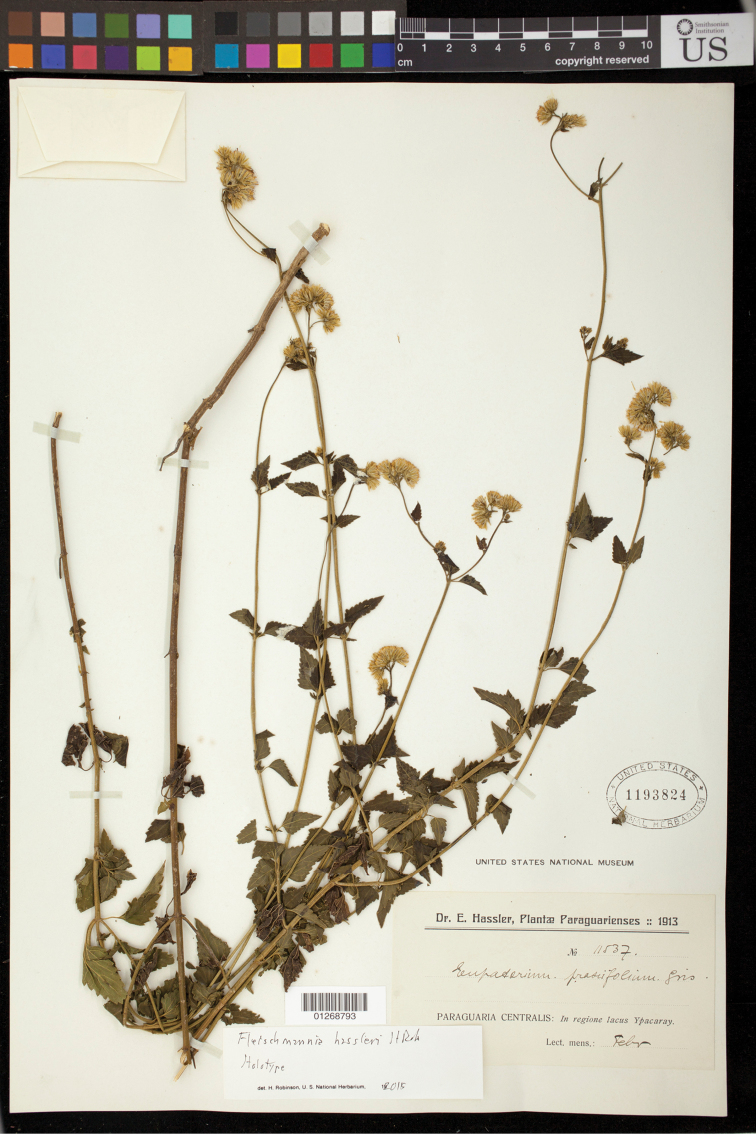
*Fleischmannia hassleri* H.Rob., holotype (US).

Paraguay: Centralis, in regione lacus Ypacaray, Feb. 1913, *Hassler, Plantae Paraguariensis 1913* (holotype US). (Figure 1)

##### Description.

Erect or leaning subshrubs to 0.6–1.0 m tall, with internodes 3–5 cm long, longer distally; with branches ascending at less than 45° angles. Leaves opposite, petioles 5–10 mm long; leaf blades triangular with subtruncate to truncate bases, mostly 2–3 cm long, 1.0–1.8 cm wide, smaller at more distal nodes, with ca., 7 teeth on each margin, apex acute, densely puberulous on both surfaces, with numerous small glandular dots abaxially, triplinervate from narrow basal acumination, secondary veins strongly ascending. Inflorescence terminal on slender elongated stems and branches, cymiform with branches ending in small clusters of heads; peduncles 1–3 mm long, densely puberulous; capitula ca. 6 mm high, ca. 4 mm wide before spreading; involucral bracts ca. 22. 1.5–4.0 mm long, 1.0–1.2 mm wide, 1 or 2 basal involucral bracts short and oval, acute, puberulous outside, median and inner bracts stramineous, membranous with scarious margins and tips, blunt, with pair of large longitudinal veins and often weak 3^rd^ or 4^th^ veins; florets 17–20 in a capitulum; corollas purple, ca. 3 mm long, basal tube ca. 0.5 mm ling, throat ca. 2 mm long, lobes ca. 0.5 mm long; anther thecae ca. 1 mm long; style branches slender, achenes ca. 1.2 mm long, ribs slightly yellow when submature, smooth except for a few small scabrae; pappus whitish, ca. 3 mm long, with ca. 32 bristles, not broader near the base.

Paratypes: Paraguay. Villamein? common in thickets, *Jorgensen 54268* (US). Dept. Cordillera, Compañia Yoypó-jú, disturbed sandy soil, with sandstone outcrops, among herbs, grasses and bushes in abandoned quarry, edge of disturbed forest. 3 March 1985, *Bordas 3669* (US). Dept. Central, Nemby (Barrio Industrial, Segunda Etafa) in advancing disturbed forest, in clearings, leaning on other plants. 13 March 1986, *Bordas 3750* (US). Dept. Central, border Tavarory-Acosta ñu, creek, affluent of Paraguay River, gallery forest, 25°27'S, 57°32'W, 10 June 1993, *E. Zardini & V. Jara 36127* (AS, MO, US). Caaguazú, Feb. 1905, *Hassler 9069* (US).

Specimens have usually been determined in the past as *Fleischmannia prasiifolia* with which it shares many characteristics such as ascending branches, short peduncles in the inflorescence, purple color of the corollas and achenes with somewhat paler ribs. The new species differs by the smaller more densely pubescent leaves and smaller clusters of heads, and it occurs far to the east of the distribution of *Fleischmannia prasiifolia* and at generally lower elevations. The presence of minute glandular dots on the abaxial leaf surfaces would also seem to be a distinction, but minute stipitate glands occur in Fleischmannia prasiifolia var. glandulifera as noted in the Bolivian treatment.

### Brazil

*Fleischmannia* in Brazil has mostly been recognized as having three species, the widespread *Fleischmannia microstemon* (Cass.) R.M. King & H. Rob., *Fleischmannia remotifolia* (DC.) R.M. King & H. Rob. and *Fleischmannia laxa* (Gardn.) R.M. King & H. Rob. The more southern species *Fleischmannia laxicephala* (Cabrera) R.M. King & H. Rob. is a more recent addition, and two additional new species are added here from Mato Grosso and a newly validated variety from coastal Brazil. The six species and one variety are distinguished as follows:

**Table:** 

1	Annual plants; with slender branches strongly ascending at less than 45° angles, often over-topping main axis	**2**
–	Perennial plants; with branches mostly spreading at 45–90° angles	**3**
2	Peduncles puberulous, leaves obtuse to slightly acuminate; ribs of semimature achenes persistently pale	**Fleischmannia microstemon var. microstemon**
–	Peduncles hispid with small stipitate glands, leaves with short abruptly acuminate tips; ribs of cypselae not persistently pale	**Fleischmannia microstemon var. paniculata**
3	Capitula in rather dense corymbiform clusters, style branches broadened apically; upper margins of inner involucral bracts often somewhat ruffled	***Fleischmannia remotifolia***
–	Capitula laxly disposed, on peduncles often 5 mm long or more, style branches not broader distally; margins of involucral bracts not ruffled	**4**
4	Pappus bristles broadened at base; peduncles densely puberulous	***Fleischmannia laxicephala***
–	Pappus bristles not broadened at base, not or scarcely contiguous with each other; peduncles, hispid with stipitate glands or nearly glabrous	**5**
5	Peduncles hispid with small stipitate glands; inner involucral bracts acute with narrow scarious borders; corollas usually lavender	***Fleischmannia laxa***
–	Peduncles glabrous or subglabrous with sparse non-glandular hairs; inner involucral bracts with rounded or obtuse tips and broadly scarious borders; corollas white	**6**
6	Leaf blades membranaceous, broadly ovate with sharply serrate margins, puberulous on veins and with scattered minute glandular dots abaxially	***Fleischmannia matogrossensis***
–	Leaf blades subcoriaceous, narrowly ovate with scarcely serrate margins, surfaces pilosulous and without glandular dots	***Fleischmannia hatschbachii***

#### Fleischmannia hatschbachii

Taxon classificationPlantaeAsteralesAsteraceae

H. Rob. sp. nov.

urn:lsid:ipni.org:names:77151274-1

##### Type.

**Figure 2.**
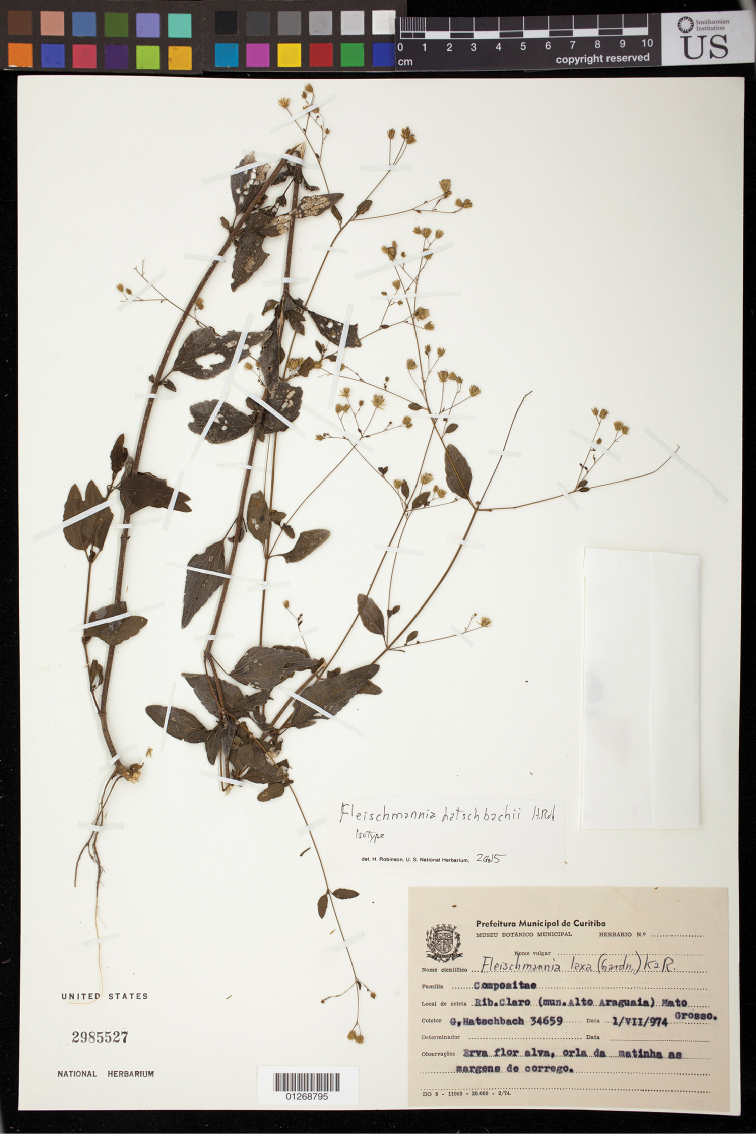
*Fleischmannia hatschbachii* H.Rob., isotype (US).

Brazil: Mata Grosso, Ribeirão Claro (mun. Alto Araguaía), 17°15'46"S, 53°11'54"W; erva flor alva, orla da matinha as margens de corrego, 1 July 1974, *Hatschbach 34659* (holotype MBM, isotype US). (Figure 2)

##### Description.

Erect herb 0.6–0.7 m tall, stems brownish, terete, striated when dry, hispid with small hairs. Leaves opposite, petioles 0.5–1.0 cm long, puberulous; blades ovate, 2.5–5.0 cm long, 1.0–1.8 cm wide, widest at basal 1/4or 1/3, base obtusely to acutely cuneate, margins bluntly subserrate with 4–6 teeth on each side, apex acute, or slightly blunt at tip, adaxial surface sparsely pilosulous; abaxial surface with prominent pale primary and secondary scabrid veins, areolae glabrous, without evident glandular dots, triplinervate from base with strongly ascending secondary veins. Inflorescence a lax panicle with mostly opposite branches spreading at mostly ca. 45° angles; mostly glabrous; main axis with foliiform bracts 0.6–2.0 cm long and 0.3–1.1 cm wide; branches filiform, peduncles 6–15 mm long, glabrous. Heads ca. 5 mm high, 3 mm wide; Involucral bracts ca. 25 in ca. 4 series, narrowly ovate to narrowly oblong, 1.0–3.5 mm long, 0.5–0.8 mm wide, with broadly scarious obtuse tips; florets ca. 17 in a head; corollas white, ca. 2.3 mm long, basal tube ca. 0.5 mm long, throat ca. 1.5 mm long, lobes ca. 0.5 mm long, with short monoseriate hairs on outer surface; anther thecae ca. 0.8 mm long; apical appendage ca. 0.3 mm long; style branches not broader at tips. Achenes submature, ca. 1.7 mm long. brownish, ribs slightly paler, setuliferous on ribs; pappus whitish, ca. 2 mm long, of ca. 25 slender scabridulous non-contiguous bristles.

Only the type collection is known.

*Fleischmannia hatschbachii* is one of two new species from Mato Grosso described here with slender glabrous or nearly glabrous branches of the inflorescence. This species and *Fleischmannia matogrossensis*, nevertheless, differ too much in their leaves to be the same species. A third species with glabrous branches of the inflorescence is found farther west in Bolivia, *Fleischmannia neei*. That differs by its less slender inflorescence branches, lavender corollas and its more glabrous leaf surfaces.

#### Fleischmannia laxa

Taxon classificationPlantaeAsteralesAsteraceae

(Gardn.) R.M. King & H. Rob., Phytologia 19: 204. 1970

Eupatorium laxum Gardn., London J. Bot. 5: 476. 1846. Type: Brazil. Minas Gerais, In bushy places on the banks of the Rio Claro, June 1841, *Gardner 4856* (holotype BM, isotype US).

##### Description.

Plants probably always perennial, scandent or scrambling stems, branches spreading at 65–80° angles. Leaves opposite; petioles 0.5–3.0 cm long; blade narrowly to broadly ovate, mostly 2–5 cm long, 1.5–4.0 cm wide, base obtuse to truncate, margins with 8–12 blunt teeth on each side, apex shortly acuminate, surfaces finely and densely hispidulous, abaxial surface paler, with scattered minute glandular dots; triplinervate from short basal acumination, major veins often pale. Inflorescence laxly branching; peduncles 5–25 mm long, hispid with stipitate glands; heads ca, 6 mm high, 4 mm wide; involucral bracts ca. 30 in ca. 4 series, 1–5 mm long, outer narrowly ovate, inner bracts narrowly oblong, inner bracts with green part reaching short-acute apices, scarious margins narrow. Florets ca. 17 in a head; corollas usually lavender or reddish, ca. 3 mm long, basal tube ca. 0.5 mm long, throat ca. 2 mm long, lobes ca. 0.5 mm long, without hairs outside; anther thecae ca. 0.8 mm long, apical appendage ca. 0.2 mm long; style branches slender. Achenes 1.5–1.8 mm long, not noticeably paler on ribs, setuliferous on ribs and sometimes on distal surfaces; pappus whitish, of ca. 20–23, slender non-contiguous bristles 2.5–2.8 mm long, not broadened at base, with dense fringe of setulae along margin of callus and between or on bases of bristles.

##### Specimens seen.

Brazil: Distrito Federal: Côrrego Samambaia, near Taguatinga, ca. 20 km W of Brasília, elev. 1000 m, Gallery forest, subshrub ca. 1.5 m tall, heads pale lavender, 13 July 1966, *H.S. Erwin, J.W. Grear Jr.*, *R. Sousa & R. Reus dos Santos 18196* (NY, UB, US); Distrito Federal: Reserva Ecológica do IBGE. Planta bastante delgada, capítulos alvacententos, beira da mata das nascentes de córrego Taquara, 23 June 1983, *B.A.S. Pereira 587* (IBGE, US); Distrito Federal: Reserva Ecológica do IBGE, mata ciliar do córrego Roncador, 15°58'06"S, 47°53'43"W, erva ca. 1.2 m de altura, botão floral de cor verde, flores de cor rosa, 12 June 1989, *D. Alvarenga & F.C.A. Oliveira 298* (IBGE, US); Goiás: Alto Paraíso, rod. Para Nova Roma, Pedra Ruim, capítulos alvos, transição campo cerrado-rupestra, 13 June 1993, *G. & M. Hatschbach 59488 & Barbosa* (MBM, US); Minas Gerais: Est. ecologica UFMG-BHMG, mata, beira da trilha, 13 July 1990, *E. Tameirão Neto 85 & Glauco* (BHCB 22.876, US); Est. ecologica UFMG-BHMG, cerrado, escandente voluvel, 16 May 1990, *E.M. Santos 25 et alii* (UFMG, US); Minas Gerais: Parque Nacional Grande Sertão Veredas, margens do rio Mato Grande, 15°119'06"S, 45°59'09"W, erva, annual, approx. 60 cm compr., capitulos arroxeados, 29 Apr. 1999, *R. Rodrigues-da-Silva, T.S. Filgueras & F.C.A. Oliveira 259* (IBGE, US).

The obvious stipitate glands on the peduncles are distinctive. In Brazil, similar glands are seen elsewhere only in Fleischmannia microstemon var. paniculatum H.Rob. described below.

#### Fleischmannia laxicephala

Taxon classificationPlantaeAsteralesAsteraceae

(Cabrera) R.M. King & H. Rob., Phytologia 19: 204. 170.

Eupatorium laxicephalum Cabrera, Sellowia 15: 196. 1963. Type: Brazil. Santa Catarina, (Mun. Lauro Müller), Ruderal, lower and middle slope of serra by Rio do Rastro, 20 km west of Lauro Müller, alt. 700–1000 m, 3-IV-1957. *L.B. Smith & R. Klein 12341* (holotype LP, isotype US).

##### Description.

Shrubs with many opposite, woody branches spreading at ca, 45° angles; stems terete, glabrous. Leaves opposite, petioles slender, 0.5–1.2 cm long, blades 1.5–5.0 cm long, 0.5–2.5 cm wide, base truncate to short-acute, margins with numerous jagged teeth, apex narrowly acute to slightly attenuate, surfaces glabrous, triplinervate from slight acumination at petiole, main veins pale. Inflorescence rather diffuse, with single or few heads at tips of leafy branches, slender glabrous peduncles 5–15 mm long, heads ca. 6 mm high; involucral bracts ca. 30, with few lanceolate outer bracts 1.0–1.5 mm long, inner bracts narrowly oblong, to 4 mm long, with mucronate tips; florets ca. 17 in a head; corollas white, ca. 2 mm long, basal tube ca. 0.5 mm long, throat ca. 1 mm long, lobes ca. 0.5 mm long, without evident hairs outside; anther thecae ca. 0.8 mm long, apical appendage ca. 0.2 mm long, style branches slender. Achenes ca. 1.2 mm long, ribs not pale, sparsely setuliferous; pappus white, ca. 2 mm long, of ca. 30 barbellate bristles, obviously broadened and contiguous at base.

**Additional specimens seen.** Brazil, Santa Catarina. Mun. Lauro Müller, Rod. SC-438, Serra do Rio do Rastro, erva, de capítulos alvos, paredões rochosos, 1000 m, 7 Apr. 1991, *G. & M. Hatschbach 55312 & D. Guimarães*, (MBM, US); Mun Lauro Müller, Serra Rio do Rastro, capítulos alvos, paredões rochosos úmidos, 18 Apr. 1994, *G. Hatschbach 60640 & E. Barbosa* (MBM, US); Serra da Rocinha, alto (Mun. Timbé do Syl), ereta, capitulo alvo, paredão roschoso, 13 March 2005, *G, Hatschbach*, *E. Barbosa & E.F. Costa 79122* (MBM, US); Serra do Rio do Rastro (Mun. Lauro Müller), ereta 35 cm, flor alva, Paredão rochoso, alt. 1100–1200 m, 16 March 2005, *G. Hatschbach,I E. Barbosa & E.F. Costa 79218* (MBM, US); Serra do Rio do Rastro (Mun. Lauro Müller), arbusto, 80 cm, capitulo alvacento, encosta de paredão úmido, 16 Nov. 2008, *J.M. Silva,I J. Cordeiro*, *C.B. Poliquesi & J. Vaz 7194* (MBM, US).

The specimens seen are all woodier with more regular vegetative branching than most members of the genus *Fleischmannia*. This is only Brazilian species known with obviously broadened bases on the pappus bristles. The species is presently known only from Santa Catarina.

#### Fleischmannia matogrossensis

Taxon classificationPlantaeAsteralesAsteraceae

H. Rob. sp. nov.

urn:lsid:ipni.org:names:77151275-1

##### Type.

**Figure 3.**
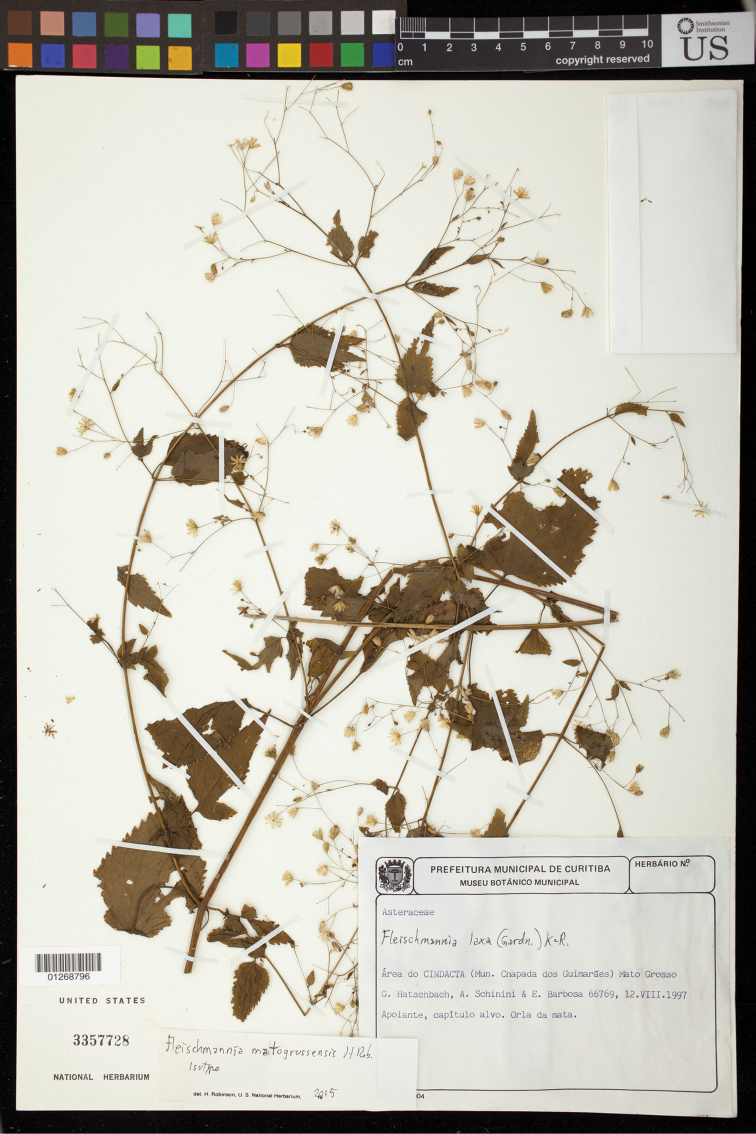
*Fleischmannia matogrossensis* H.Rob., isotype (US).

BRAZIL: Mato Grosso, Area do Cindacta (Mun, Chapada dos Guimarães), 15°27'39"S, 55°45'W Apoiante, capitulo alvo, Orla da mata, 12 VIII 1997, *G. Hatschbach, A. Schinini & E. Barbosa 66769* (Holotype MBM, isotype US; see Figure 3).

##### Description.

Scrambling vines 2 or more meters long, stems brown, striated when dry, densely puberulous below, becoming sparsely pilosulous distally, glabrous in inflorescence. Leaves opposite, petioles 0.5–2.3 cm long, puberulous; blades membranous, broadly ovate, below inflorescence mostly 5–7 cm long, 3.0–4.5 cm wide, widest near basal 5^th^, base subtruncate, margins sharply toothed with 12–20 teeth on each side, apex usually gradually narrowly acuminate, adaxial surface subglabrous, abaxial surface concolorous, puberulous on primary and secondary veins, glabrous between, with scattered minute glandular dots, triplinervate from petiole. Inflorescence a lax pyramidal panicle with slender opposite branches spreading at 45–80° angles, main axis with foliiform bracts up to 3 cm long, with 5–8 serrations on each margin; branching laxly cymiform distally; peduncles filiform, mostly 8–12 mm long, glabrous. Heads ca. 6 mm high and 3–4 mm wide, with ca. 25 bracts in 4–5 series, mostly stramineous, outer bracts ovate, 1–2 mm long, inner bracts narrowly oblong to linear-oblong, to 4.5 mm long, 0.8–1.0 mm wide, with scarious margins and obtuse scarious tips; receptacle scarcely convex. Florets ca. 17 in a head; corollas white, ca. 2.5 mm long, basal tube ca. 0.5 mm long, throat ca. 1.5 mm long, lobes ca. 0.45 mm long; anther thecae ca. 0.8 mm long, apical appendage ca. 0.3 mm long. Achenes ca. 1.8 mm long, black at maturity, with short setulae on ribs; pappus white, ca. 2 mm long, with ca. 25 scabridulous bristles, scarcely broadened near base, not or scarcely contiguous.

The species is known only from the type collection. In general habit, the species seems closest to *Fleischmannia laxa*, but it has no stipitate glands on the branches of the inflorescence as seen in the latter species, the leaves are not densely hispidulous with margins more sharply toothed and the involucral bracts are less pointed with more broadly scarious margins.

#### Fleischmannia microstemon (Cass.) R.M. King & H. Rob. var. microstemon

Taxon classificationPlantaeAsteralesAsteraceae

, Phytologia 19: 204. 1970.

Eupatorium microstemon Cass., Dict. Sci. Nat. 25: 432. 1822. Weed in Paris botanical Garden, *Cassini s.n.* (holotype P).
Eupatorium guadaloupense Spreng., Syst. Veg. 3: 414. 1826. Guadeloupe, *Bertero s.n.* (P?, TO?)
Eupatorium bimatrum Standl. & L.O. Williams, Ceiba 3: 64. 1952. Honduras, Morazán, El Zamorano, ca. 800 m, 18 Oct. 148, *Standley 13132* (holotype F).

##### Description.

Erect annual herbs to 1 m tall, with slender, strongly ascending branches which often overtop the main axis; stems yellowish green to brown, puberulous. Leaves mostly opposite, alternate distally; petioles slender, usually 0.8–4.0 cm long; leaf blades membranaceous, broadly rhombic-ovate, mostly 2.0–3.7 cm long, 1.4–3.0 cm wide, base acuminate at petiole, margins crenate with 7–14 teeth on each side, apex usually short-acute, adaxially sparsely pilose, abaxially pilosulous on veins, with glandular dots; triplinervate from margins of basal acumination. Inflorescence lax cymose panicles with ascending branches, with many shorter lateral branches proximally; peduncles 2–7 mm long, slender typically puberulous. Heads ca. 4 mm high; involucral bracts 15–22 in 3–4 series, 1.5–3.8 mm long, outer narrowly acute, sparsely puberulous, inner bracts broad and mostly scarious at tip, apex apiculate. Florets 20–35 in a head; corollas lavender to white, 2.0–2.2 mm long, basal tube ca. 0.5 mm long, throat ca. 1.5 mm long, lobes ca. 0.2 mm long, without hairs outside; anther thecae ca. 0.7 mm long; style branches narrowly linear. Achenes 1.3–1.5 mm long, ribs persistently yellow, ribs and distal faces setuliferous; pappus white, of ca. 20 slender not or scarcely contiguous bristles ca. 2 mm long.

#### Fleischmannia microstemon var. paniculata

Taxon classificationPlantaeAsteralesAsteraceae

H. Rob. var. nov.

##### Type.

**Figure 4.**
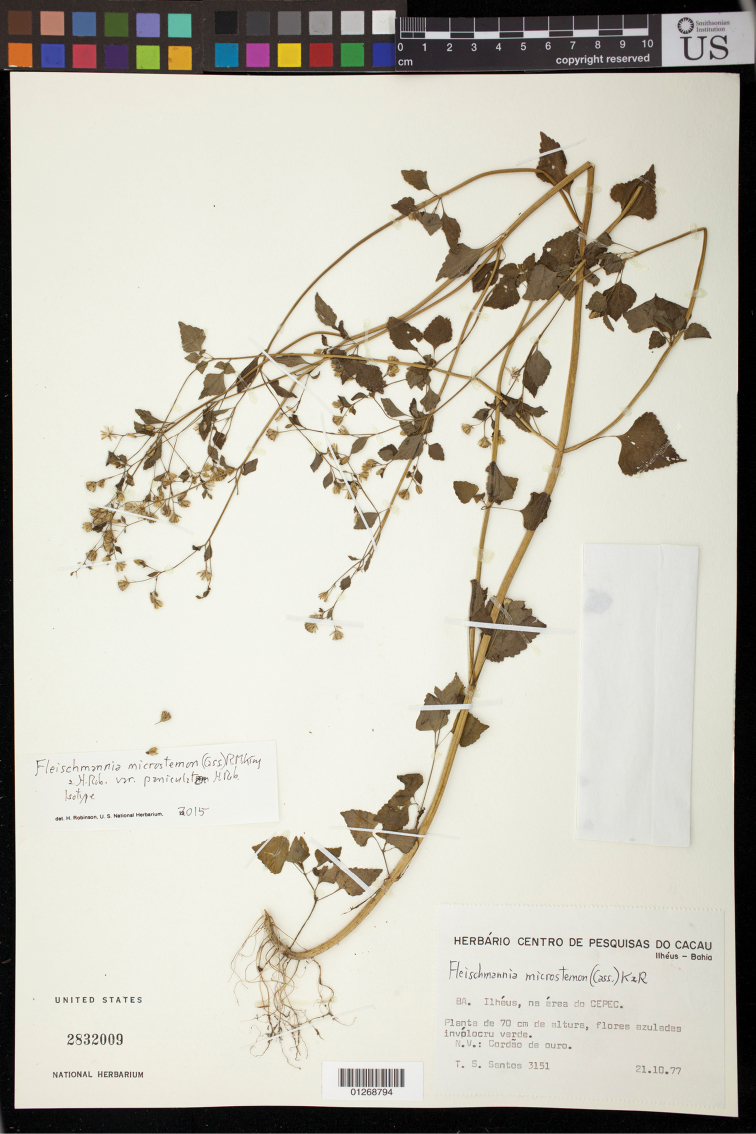
Fleischmannia microstemon var. paniculata H.Rob., isotype (US).

Brazil, Bahia, Ilhéus, ns área do CEPEC, Plants de 70 cm de altura, flores azuladas, invólucru verde, N.V. Cordão de Ouro, 21.10.1977, *T.S. Santos 3151* (holotype CEPEC, isotype US). (Figure 4).

?*Eupatorium paniculatum* Schrad., Ind. Sem. Hort. Acad. Gott. 2. 1832; Linnaea 8, Litt. 26. 1833, hom. illeg., non Miller 1768. Photo from G-DC. Herbarium seen.

With the habit of the typical variety, but differing by the rather abrupt short acuminatie tips on leaf blades, stipitate glands on the peduncles and branches of the inflorescence, and by the not or scarcely paler ribs on the submature achenes.

##### Paratypes.

Brazil: Bahia, 1842, *Glocker no 18* (BM, US); Bahia, forests of the Rio Grongogy Basin, alt. 100–500 m, 1 Oct.–30 Nov. 1915, *H.M. Curran 147* (US); Paraiba do Norte, Areias; Escola de Agronomia do Nordeste, herva colluda em logares relativamente umidos, 9–3-1947, *J.de Moraes Vase 17* (RB, US); Pernambuco, Tapera, at wet shady border of a thicket, 26 July 1931, *D.B. Pickel s.n.* (LCU, US).

The variety is evidently restricted to areas near the coast of northestern Brazil. The locality in Bahia and the shape of the leaf blades as seen in the photograph of the G-DC specimen make it almost certain the Schrader nom. illeg. represents this same variety. While there is no nomenclatural requirement for perpetuating the Schrader name it has been decided to do so.

#### Fleischmannia remotifolia

Taxon classificationPlantaeAsteralesAsteraceae

(DC.) R.M. King & H. Rob., Phytologia 19: 205. 1970

Fleischmannia remotifolium DC., Prodr. 5: 165. 1836.

##### Type.

Brazil. Minas Geraes near Mariannum, *Vauthier 259* (lectotype G-DC).

Specimens are usually described as vines. Stems have branches usually spreading at ca. 90° angles. The leaves have mostly 9 to 14 blunt or sharp teeth on each margin, and vary from ovate with acute apices to narrowly ovate or lanceolate with narrowly acuminate apices. Inflorescence with elongate panicle with widely spreading branches, heads closely clustered at ends of branches; peduncles 2–6 mm long, puberulous. Heads ca. 7 mm high, 4–5 mm wide; involucral bracts ca. 22 in ca. 4 series; all bracts with acute to acuminate tips, 2–6 mm long, outer bracts narrowly ovate, inner bracts narrowly oblong. Corollas are rather consistently described as white, but sometimes lavender, ca. 3 mm long, basal tube ca. 0.5 mm long, throat ca. 2 mm long, lobes ca. 0.4 mm long, glabrous outside; anther thecae ca. 0.9 mm long, apical appendage ca. 0.25 mm long; style branches broadened distally. Achenes ca. 2 mm long, not paler on ribs, weakly setuliferous on ribs; pappus whitish, of ca. 35 bristles ca. 3 mm long, slightly broadened and contiguous at base, often detaching in groups.

##### Specimens seen.

Brazil: Bahia: mun. Arataca, Serra das Lontras 9.0 km WNW of Itatinguí on Fazenda road, then on trail over mountain ridge to Dazenda Cairo, 15°11'53"S, 39°23'50"W, 530–630 m, disturbed southern Bahian moist forest, fallen liana, mostly leafless, involucre olive, heads white, 15 Sept. 2004, *W.W. Thomas, A.M. Amorim, J.G. Jardim, S. Sant’Ana & J.L. Paixão 14062* (NY, US). Bahia-Espirito-Santo: Vale do Rio Mucurí, ao lado de Rodovia BR 101, mata de terra firme, planta e 2 m de altura, capítulos brancos, involucro verde, 16 July 1968, *R.P. Belem, 3873* (CEPLAC, NY, US); Espirito Santo, Macuco, Reserva de Scoratama, mata alagadiça de palmetto, ca.70 cm de alt,, ramos herbaceous e decumbentes, semi-umbrofila, capitulos roseo-cloros, 16 Aug. 1969, *D. Grew 5642* (RB, US); Espirito Santo: Rod. BR-262 (mun. Ihatiba, erva de flor alva, capoeira, local sombrio, úmido, 21 July 1982, *G. Hatschbach 45185 & C. Guimarães* (MBM, US); Espírito Santo, São Bento de Urâmia, rodovia para Castelinho (mun. Alfredo Chaves), apoiante, capítulos lilás, Orla da mata plubial de solo arenoso, alt. 900 m, 8 Oct. 1994, *G. & M. Hatschbach 61153 & S.M. Silva* (MBM, US); Espirito Santo: Reserva Florestal de Linhare, Linhares, km. 1.5, Aceiro com Ceolin, Vatzea periodicimente inundável, Rastejante, herbáceo, flor branco, exudação seiva, incolor, common name Arnica falsa, 29 July 1998, *D.A. Folli 3203* No. registro 5955 (CVRD, US); Minas Gerais: Caldas, 1845, *Widgren 187* S, US); Minas Gerais: 18 July 1862, *Regnell I232* (S, US); Minas Gerais: Agricultural College lands, Chacha Valley, tangle on overgrown eastern slope, scandent shrub 2.5 m high, with long branches, white flower, infrequent, 30 June 1930, *Ynes Mexia 4818* (US); Minas Gerais: Viscosa, Fazenda de Faziuma, forest on slope, in partial shade, alt. 700 m, suffrutescent, scandent, 1.5 m high, white flower with pungent fragrance, occasional. 8 Sept. 1930, *Ynes Mexia 4944* (US); Minas Gerais, Distrito Ilheu, Fazenda da Tabunha, trail to Capichava, in partial shade in cut-over woods, vine climbing 3 m high, flower white, agreeably pungent odor, alt. 210 m, 30 Aug. 1930, *Ynes Mexia 5019* (US); Rio de Janeiro: alt. 21 ft?, *Miers 3719* (BM, US); Minas Gerais: Mata, Serra do Curral, mun, de Belo Horizonte, pouco frequente, flores alvas, arbusto semiescandente, 25 July 1942, *M. Magalhães 3304* (SP, US); Minas Gerais: Parque Nacional do Cachoeira Bonita (Mun. Caparaó), erva 50 cm, capitulos alvos, matinha de alt. 1800 m, 15 June 1991, *G. & M. Hatschbach & D. Guimarães 55534* (MBM, US); São Paulo: (capital), on margin of a rivulet, white flowers, *B. Prokel 5333* (US); São Paulo, aguas de Lindois, semi-prostrada, á sombra da mata, á beira de córrego, corolas alvas, 17 Aug 1968, *W. Hoehne 6248* (SP, US)

This is the most common species of *Fleischmannia* in eastern Brazil. It is distinct from other Brazilian species by the more densely corymbiform inflorescence and broader style branches.

### Bolivia

The understanding of *Fleischmannia* in Bolivia has been particularly bad, with most of the specimens misidentified. The key to the Bolivian species of *Eupatorium* by B.L. Robinson (1920), treated the species intermixed with other species that were included in *Eupatorium* at the time. It relied on characters such as number of flowers in the heads which are useful in other elements then known in *Eupatorium* but usually not in *Fleischmannia*. It was also based on extremely inadequate collecting, especially in southern Bolivia. The Robinson (1920) treatment did not include the Argentinian species, *Eupatorium prasiifolium* Griseb. and *Eupatorium schickendantzii* Hieron., which are now known to occur in southernmost Bolivia. Among material previously misidentified by the present author are specimens here described as two new species, and some other individual collections have been seen that suggest still other undescribed species of the genus occur in Bolivia. Certainly, more collections of members of the genus in Bolivia is necessary.

Characteristics that seem more reliable at the species level in *Fleischmannia* are the habit of the plants, the angle of branching, the pubescence, density of the inflorescence, width of the style branch tips, the basic color of the corollas and the width of the bases of the pappus bristles.

Twelve species are known from the country including two that also occur in Argentina and the widely distributed *Fleischmannia microstemon*. One species that has been credited to Bolivia, *Fleischmannia marginata* (Poepp.) R.M. King & H. Rob. is actually found only the Dept. Junin in central Peru. The species cited in most previous studies such as Robinson (1920) as *Eupatorium pycnocephalum* Less. from southern Central America and South America are actually *Fleischmannia pratensis* (Klatt) R.M. King & H. Rob.

No material has been seen in this study that perfectly matches the descriptions of *Fleischmannia bridgesii*, *Fleischmannia fiebrigii* or *Fleischmannia tamboensis*. Their positions in the following key are based on descriptions by original authors, and B.L. Robinson (1920) plus a few specimens that may be *Fleischmannia bridgesii* seen in this study.

The species of *Fleischmannia* now known from Bolivia are distinguished as follows:

**Table:** 

1	Peduncles and often other parts of plants with stipitate glandular pubescence	**2**
–	Plants without stipitate glandular hairs	**5**
2	Plants with branches strongly ascending, spreading at less than 45° angles, usually without reddish hairs; corollas lavender to reddish colored	**3**
–	Plants often scrambling or scandent with branches spreading at 45–90° angles, often with reddish hairs; corollas lavender to white	**4**
3	Nearly erect plants with dense cover of minute stipitate glands on stems, leaves and peduncles; leaves acute; inflorescence with dense corymbiform clusters of many heads	**Fleischmannia prasiifolia var. glandulifera**
–	Erect to recumbent plants covered with viscous stipitate glands; leaf tips mostly short acute or obtuse; inflorescence with loose clusters of few heads	***Fleischmannia fiebrigii***
4	Leaves scabridulous on adaxial surface, scabrid on veins abaxially; corollas with the limb lilac	***Fleischmannia tamboensis***
–	Leaf blades covered with stipitate or viscid glandular hairs; corollas white	***Fleischmannia yungasensis***
5	Plants annual, with filiform and erratically bent ascending lateral branches spreading at less than 30° angles, some over-topping the primary stem	***Fleischmannia microstemon***
–	Perennial herbs, with branches, when present, well-developed, ascending to widely spreading, usually not over-topping the the primary stem	**6**
6	Inflorescence with heads in large rather dense corymbiform to rounded or pyramidal clusters	**7**
–	Heads few or single at tips of elongate branches	**10**
7	Corollas white	***Fleischmannia polopolensis***
–	Corollas pink to lavender or purplish distally	**8**
8	Involucral bracts mostly lanceolate; petioles short, less than 1/5 as long as leaf blade; material seen under this name without branches below inflorescence	***Fleischmannia bridgesii***
–	Involucral bracts mostly oblong; petioles long and slender; with branches spreading at less than 30–60° angles	**9**
9	Pappus bristles slender at base, often not contiguous; achenes with pale ribs	***Fleischmannia prasiifolius***
–	Pappus bristles broader at base, contiguous; achenes without pale ribs	***Fleischmannia pratensis***
10	Heads with ca. 7 florets and 20 involucral bracts; leaves lanceolate; with few sharp teeth on margins; peduncles mostly over 2 cm long	***Fleischmannia soratae***
–	Heads with 17–25 florets and 30 or more involucral bracts; Inflorescences terminating in widely spreading cymiform branches	**11**
11	Peduncles and branches of inflorescence glabrous or subglabrous; corollas ca. 2 mm long; anther thecae ca. 0.5 mm long	***Fleischmannia neei***
–	Peduncles and branches of inflorescence puberulous; corollas ca. 3 mm long; anther thecae 1–2 mm long	**12**
12	Branching in inflorescence opposite to near ultimate nodes; peduncles 0.3–0.4 mm wide; inner involucral bracts obtuse or mucronate with rather broadly scarious tips; corolla lobes with styliform papillae on outer surface; achenes without noticeably paler ribs	***Fleischmannia steinbachii***
–	Branching in inflorescence often subopposite or alternate at distal nodes; peduncles filiform, 0.1–0.2 mm wide; inner involucral bracts acute with narrowly scarious tips; corollas without hairs or styliform papillae on outer surface; achenes with pale ribs	***Fleischmannia schickendantzii***

#### Fleischmannia bridgesii

Taxon classificationPlantaeAsteralesAsteraceae

(B.L. Rob.) R.M. King & H. Rob., Phytologia 19: 203. 1970

Eupatorium bridgesii B.L. Rob., Proc. Amer. Acad. 55: 7. 1919. Type: Bolivia, without specific locality, *Bridges s.n.* (holotype K, photo GH).

##### Description.

Perennial herbs to 60 cm high; stems brownish to reddish-tinged, terete, densely pilosulous, without known branches but often with axillary fascicles. Leaves opposite, petioles, short, 4–7 mm long, a 5^th^ as long as blade; leaf blade narrowly ovate, with pale main veins, Inflorescence rather dense, flattened corymbiform; branches strongly ascending, puberulous; peduncles 3–7 mm long, puberulous. Heads ca. 6 mm high and 4 mm wide; involucral bracts ca. 22, gradate, 1.5–5.0 mm long, described as lanceolate, attenuate, most specimens seen with bracts more oblong and less pointed, often reddish distally. Florets ca. 25 in a head; corollas pink, 3–4 mm long, basal tube ca. 0.5 mm, throat 2–3 mm, lobes ca. 0.5 mm long, without evident hairs; anther thecae ca. 1 mm long, apical appendage ca. 0.25 mm long; style branches not broadened distally. Achenes ca. 2 mm long, with paler ribs, pappus setae white, ca. 27, 2.5–3.0 mm long, slender, scarcely contiguous.

Specimens seen that bear the name, have thin pale-green leaves with slightly paler abaxial surfaces and white main veins. The petioles in all four specimens are comparatively short, 1/5 the length of the blade or less. They are unbranched with flat-topped corymbiform inflorescences with strongly ascending inflorescence branches, and have at least the outer involucral bracts lanceolate. These specimens do not show the squarrose-spreading tips of the outer involucral bracts cited by Robinson (1920), but this characteristic is probably only a feature of an individual specimen. Such recurved tips have been seen in occasional specimens of other species as a result of something done during preparation.

Four different specimens seem to share these general characteristics. The one that seems to fit the concept best is from Bolivia: Cochabamba, 2–3 ft., 7, March 1920, *E.W.D. & Mary M. Holway 373* (US ex hb. Gray). Three additional specimens that have been determined as *Fleischmannia bridgesii*, but differ from that species in some details, are Bolivia: Cochabamba, 2600 m, 1932, *Bro Julio II 263* (US); Bolivia: Dpto Chuquisaca, Prov, Azurduy, Azurduy-Icla, arbusto, flor lila, May 1981, *E.E.B. (ERTS) 313a* (LPB, US); Chuquisaca, Sucre, alt. 2700 m, herb, ca. 50 cm high, fls. pink, April 1933, *M. Cardenas 494* (US ex hb. Gray as *Eupatorium prasiifolium*. The latter three specimens have most involucral bracts with more obtuse and more extensive reddish puberulence on stems, branches of the inflorescence and involucral bracts. All four specimens seem to be snatchings from tops of plants, and the total habit and any vegetative branching is unknown.

#### Fleischmannia fiebrigii

Taxon classificationPlantaeAsteralesAsteraceae

(Hieron.) H. Rob. comb. nov.

urn:lsid:ipni.org:names:77151290-1

Eupatorium fiebrigii Hieron. in Urban, Bot. Jahrb. Syst. 40: 371. 1908. Type: Bolivia, Tarija, Prov. Arce, in fields near Camacho, alt. 2700 m, in arvis, 15 Dec. 1903, *Fiebrig 3528* (holotype B, destroyed. photo US).

##### Description.

Perennial herbs ca. 0.6 m tall; stems pale whitish-green, striated, densely viscid glandular, glabrate; internodes to 7 cm long; branches spreading at ca. 30° angles. Leaves opposite; petiole 0.5–1.0 cm long, densely viscid-glandular; leaf blades membranaceous, pale green, broadly ovate, to 3.5 cm long, 2.5 cm wide, widest ca. 5 mm above base, base rounded to subcordate, margins crenate-serrate, each with 4–20 teeth, teeth mucronulate, apex short-acute to obtuse, surfaces with veins glandular-puberulous; triplinervate from petiole. Inflorescence with long leafy branches bearing small clusters of heads in corymbiform cymes, peduncles viscid glandular; involucral bracts 19–21, gradate, outer bracts ovate, ca. 2.5 mm long, inner bracts linear-lanceolate, with tips often lavender, acutish, sparsely glanduliferous. Florets 20–25 in a head; corollas ca. 4 mm long, lilac, glabrous, lobes ca. 0.5 mm long, ovate-deltoid; style branches not or scarcely thickened distally. Achene ca. 1.75 mm long, angles yellowish-white, scabrous; pappus white, of ca. 20 slender bristles ca. 3 mm long, not contiguous.

The ascending branches, viscid glandular pubescence and lavender colored corollas should distinguish the species. No specimens have been seen matching this description. The strongly ascending branches indicate relationship to *Fleischmannia prasiifolia*, and some specimens of *Fleischmannia prasiifolia* placed in this study in the *var. glandulifera* also have glands. However, the specimens determined below as Fleischmannia prasiifolia var. glandulifera have very minute glands, much denser clusters of heads in the inflorescence, have petioles of the leaves much shorter and tips of the leaves narrowly acute. Further collecting in southern Bolivia should discover new material of this species, and a neotype can be established.

#### Fleischmannia microstemon

Taxon classificationPlantaeAsteralesAsteraceae

(Cass.) R.M. King & H. Rob., Phytologia 19: 204. 1970

Eupatorium microstemon Cass., Dict. Sci. Nat. 25: 432. 1822. *Cassini s.n.* (holotype P).
Eupatorium guadaloupense Spreng., Syst. Veg. 3: 414. 1826. Guadeloupe, *Bertero s.n.* (P?, TO?).
Eupatorium bimatrum Standl. & L.O. Williams, Ceiba 3: 64. 1952. Honduras, Morazán, El Zamorano, ca. 800 m, 18 Oct. 148, *Standley 13132* (holotype F).

##### Note.

See description in Brazilian treatment.

Specimens with typical obtuse to short-acute leaf shapes have been seen from higher elevations in La Paz. Specimens with more acuminate leaf tips has been seen from elevations of 500 m or less in Cochabamba and Santa Cruz.

#### Fleischmannia neei

Taxon classificationPlantaeAsteralesAsteraceae

H. Rob. sp. nov.

urn:lsid:ipni.org:names:77151276-1

##### Type.

**Figure 5.**
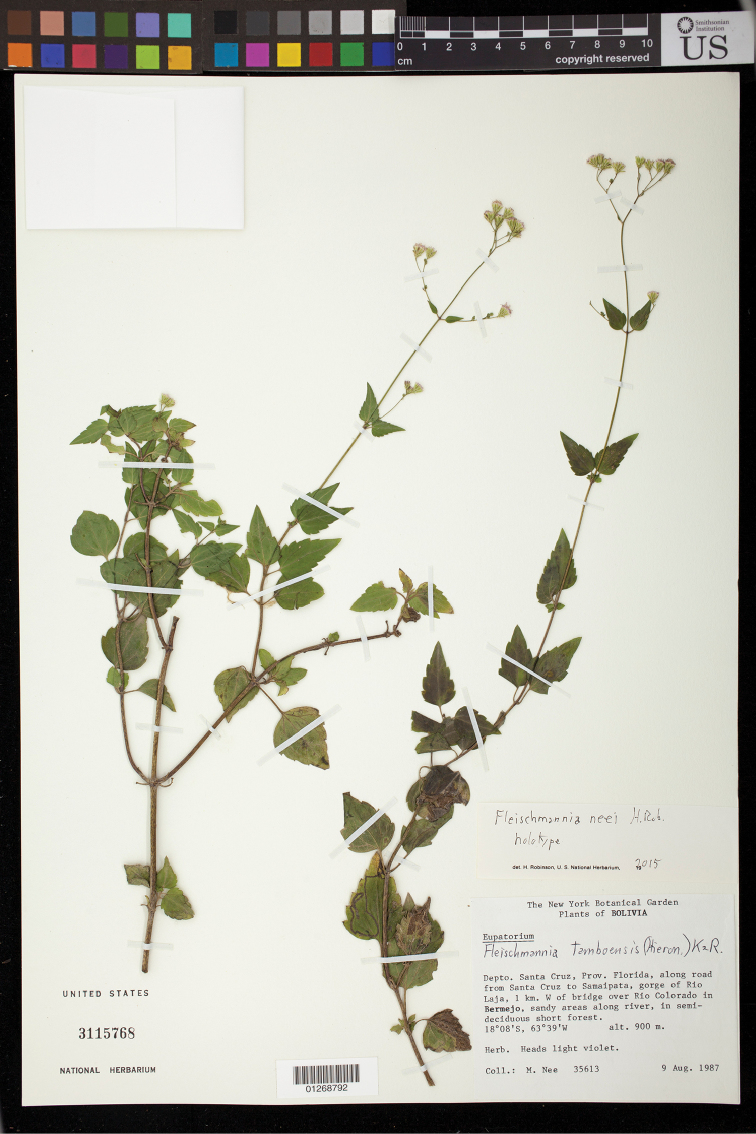
*Fleischmannia neei* H.Rob., holotype (US).

Bolivia: Santa Cruz, Prov. Florida, along road from Santa Cruz to Samaipata, gorge of Río Laja, 1 km W of bridge over Río Colorado in Bermejo, sandy areas along river, in semi-deciduous short forest, 18°08'S, 63°19'W, alt. 900 m, herb, heads light violet, 9 Aug 1987, *M. Nee 35613* (NY, US). (Figure 5).

##### Description.

Erect to scrambling perennial herbs to 1.2 m tall, stems terete, scarcely striate when dry, weakly puberulous to glabrate or glabrous; branches spreading at ca. 45 to nearly 90° angles; leaves opposite; petioles slender, 0.3–1.7 cm long, finely puberulous; leaf blades ovate, 2.3–6.5 cm long, 1–3 cm wide, base broadly rounded, scarcely acuminate at petiole, margins of lower leaves with teeth often sharply acute, apex short to narrowly acute, adaxial surface mostly glabrous, slightly shiny, abaxial surface slightly paler, puberulous on larger veins, mostly glabrous between; triplinervate from base of blade, larger veins whitish. Inflorescence a lax pyramidal panicle with mostly elongate, opposite, spreading branches, bearing small clusters of heads at tips; below and in inflorescence with small foliiform bracts on main axis; axis and branches of inflorescence glabrous or nearly so; peduncles 3–7 mm long, glabrous. Heads narrowly to broadly campanulate, 6 mm high, ca. 4 mm wide; involucral bracts ca. 25 in ca. 5 series, gradate,1–4 mm long, 0.3–1.0 mm wide, with narrowly scarious margins and apex, most basal bracts narrowly ovate, inner bracts oblong with obtuse apices, outer surfaces glabrous; florets ca. 17 in a head, corollas pale violet or pink, 2.0–2.3 mm long, basal tube ca. 0.4 mm long, throat 1.5–1.7 mm long, lobes ca. 0.3 mm long with few uniseriate hairs outside; anther thecae ca. 0.5 mm long, apical appendage ca. 0.15 mm long; style branches not broader distally. Achenes ca. 1.8 mm long, ribs sparsely setiferous above scabrid below, not or scarcely paler; pappus white, bristles ca., 30, ca. 2 mm long, not broader at base, not contiguous, fragile,

Paratypes: Bolivia: La Paz, Noryungas, Polo-Polo bei Coroico, alt. 1100 m, Oct-Nov 1912, *Otto Buchtien 3934* (US); La Paz, Prov. Nor Yungas, 4.5 km below Yolosa, then 0.7 km W on road to Río Huarinilla, 16°12'S, 67°45'W, elev. 1200 m, ford across Río Coroico, corollas lavender, 14 Nov 1982, *J.C. Solomon 8909* (MO, US); Santa Cruz, Prov. Ichilo, Parque Nacional Amboró, ca. 15 km (SE) up the Río Pitasama from the Río Surutú, moist tropical forest on lower montane slopes, sandstone, elev. 700 m, 17°44'S, 63°40'W, corollas pink, growing on dry, grassy cliff face, 28 Aug. 1985, *J.C. Solomon & S. Urcullo 14129* (MO, US); Santa Cruz, Prov. Florida, 10 km (by road) W of Bermejo, on road from Santa Cruz to Samaipata, brushy hillsides, grazed, with semi-evergreen forest, 18°09'S, 63°42'W, alt. 1150 m, 1 m tall, flowers pale violet. 6 Aug. 1987, *M. Nee & C. Coimbra S. 35527* (NY, frag. US); Santa Cruz, Prov. Florida, steep slopes with semi-deciduous forest, along nearly dry tributary to Río Bermejo, 1.5 km NW (upstream) from junction with Río Piojeras to form the Río Piraí, 18°11'S. 63°34'W, alt. 800 m, herbs to 1.2 m tall, leaves shiny above, buds only or a few pale lavender flowers, 13 July 1994, *M. Nee 45188* (NY, US); Santa Cruz, Prov. Florida, slopes of massive red sandstone along highway from Santa Cruz to Samaipata, 6.2 km (by road) from bridge over Río Laja, 18°09'S, 63°43'W., alt. 1230 m, herb, flowers light lavender, 12 July 1998, *M. Nee & D. Atha 50086* (NY, US).

*Fleischmannia neei* is most distinct among Bolivian members of the genus by the glabrous branches of the inflorescence. The *Buchtien* collection was annotated by Rusby as *Eupatorium polopolense* B.L. Robinson, probably because of the locality. Many of the other specimens were subsequently also given that name. Typical *Fleischmannia polopolensis* differs obviously in its large denser corymbiform inflorescence and its densely puberulous peduncles. Relationship of the new species might seem much closer to the newly described *Fleischmannia steinbachii*, with which it cooccurs in Santa Cruz, but the latter again has densely puberulous peduncles, more pubescent leaf surfaces, and lacks larger acute teeth on the margins of the lower leaves. An additional distinction of the new species seems to be the shorter corollas and much shorter anther thecae.

The new species also resembles the newly described *Fleischmannia hatschbachii* of Mato Grosso, Brazil, but that has thinner branches in the inflorescence, more pilosulous leaves and white corollas.

#### Fleischmannia polopolensis

Taxon classificationPlantaeAsteralesAsteraceae

(B.L. Rob.) R.M. King & H. Rob., Phytologia 19: 205. 1970

Eupatorium polopolense B.L. Rob., Contrib. Gray Herb. 61: 10. 1920. Bolivia: La Paz. North Yungas, Polo-polo near Coroico, alt. 1100, m, *Buchtien 429* (holotype GH, isotypes NY, US).

##### Description.

Perennial suffruticose herbs ca. 0.5 m tall; stems erect, terete, purplish or brownish, ca. 3 mm wide, glabrous; internodes to 10–13 cm long; branches ascending, leafy. Leaves opposite; petioles 6–13 mm long, slightly villous-puberulous; leaf blades membranaceous, ovate, 3.6–5.0 cm long, 1–2 cm wide, base rounded, slightly acuminate at petiole, margins sharply serrate with 9–18 teeth on each side 1.0–1.8 mm high, apex acuminate, both surfaces green, puberulous on veins, scarcely paler abaxially. Inflorecsnce with terminal corymbiform trifid cymes, mostly 3–5-headed, branches and peduncles, densely puberulous, non-glandulferous; peduncles short. Heads 7 mm high, 5 mm wide; involucre campanulate, with ca. 20 bracts in ca. 3 series, oval to oblong. Florets 24–30 in a head; corollas white, ca. 3 mm long, basal tube ca. 0.5 mm long, throat ca. 2 mm long, lobes ca. 0.5 mm long, hispidulous; anther thecae ca. 0.8 mm long, apical appendage ca. 0.15 mm long; style branches filiform-clavellate. Achenes ca. 1.6 mm long, black with paler ribs, ribs sparsely setuliferous; pappus white, of ca. 22 slender bristles, ca. 3 mm long, slightly broadened and scarcely contiguous at base.

##### Additional specimens.

Bolivia: Cochabamba, Sailapata, Ayopaya, 2700 m, at wet soil, 1 m, Oct 1935, *Cárdenas 3271* (US); La Paz: Nordyungas, Polo-Polo bei Coroico, alt. 1100 m, region subtropical, Oct.-Nov. 1912, *O. Buchtien 3933* (GH, NY, US).

This species includes the material that B.L. Robinson (1920) treated under the name *Eupatorium marginatum* Poepp. The supposed difference in number of florets in the heads proves spurious. The specimens cited by B.L. Robinson, were all collected by Buchtien in the same locality and share all the same obvious details. The species now known as *Fleischmannia marginata* (Poepp.) R.M. King & H. Rob. does not occur in Bolivia, but is restricted to the area of Junin in central Peru (Robinson 2001). It is distinct in its thicker leaf blades with smooth glabrous adaxial surfaces,

#### Fleischmannia prasiifolia

Taxon classificationPlantaeAsteralesAsteraceae

(Griseb.) R.M. King & H. Rob., Phytologia 19: 205. 1970

Eupatorium prasiifolium Griseb., Goett. Abh. 19: 119. 1874. Type: Argentina. Tucuman: in pratis alpinia pr. Cienega, 1873, *Lorentz 408* (holotype M).

##### Description.

Erect to scrambling perennial herbs 30–100 cm tall, stems terete, striate, drying brownish, densely hirsutulous; branches ascending, spreading at less than 30° angles, internodes 4–12 cm long. Leaves opposite; petioles 0.1–1.2 cm long; leaf blades ovate, base obtuse to subtruncate, margins each with 5–8 usually blunt serrations, apex usually narrowly acuminate, surfaces pilosulous, coarser on veins below; triplinervate from petiole, veins dark. Inflorescence of large dense clusters of heads at tips of leafy branches, branches puberulous; peduncles 2–4 mm long, densely puberulous. Heads ca. 5 mm high, 4–5 mm wide; involucral bracts ca. 30, gradate, 1.5–5.0 mm long, outer narrowly ovate, inner narrowly oblong, apices obtuse, often lavender tinged. Florets ca. 25–30 in a head; corollas reddish, ca. 3 mm long, basal tube ca. 0.7 mm long, throat ca. 1.5 mm long, lobes ca. 0.5 mm long, few or no short hairs outside; anther thecae ca. 0.8 mm long; style branches slightly broadened distally. Achenes ca. 2 mm long; with pale setuliferous ribs; pappus white, with ca. 40 bristles ca. 2 mm long, slightly broader and contiguous at base.

The most significant distinctions are the strongly ascending branches, the rather sparsely pilosulous adaxial surfaces of the leaves, the densely corymbiform clusters of numerous heads in the inflorescence, the lavender or reddish corollas and the pale ribs on the submature achenes.

##### Specimens seen.

Bolivia: Tarija, Prov. Gran Chaco, Sanadita, 90–1.20 m de altura, flor lila, 8.8.1981, *R.E.(ERTS) 294* (LPB, MO); Tarija, Prov. Cercado, 54.9 km E of Tarija-Padcaya road, on road to Entre Rios, Elev. 2050 m, Moist shrubby vegetation, heavily disturbed, 21°29'S. 64°20'W, corollas pinkish-purple, 1 May 1983, *J.C. Solomon 10306* (MO, US); Prov. Cercado, 10 km NW of Tomatas (5 km N. of Tarija), on road through Erquis, elev. 2200 m, Angusturas de Erquis, isolated packet of moist shrubby vegetation, 21°28'S, 64°50'W, corollas purplish-pink, 9 May 1983, *J.C. Solomon 10601* (MO, US).

#### Fleischmannia prasiifolia var. glandulifera

Taxon classificationPlantaeAsteralesAsteraceae

(R.E. Fries) H. Rob. comb. nov.

urn:lsid:ipni.org:names:77151291-1

Eupatorium prasiifolium var. glanduliferum R.E. Fries, Nova Acta Regiae Soc. Sci. Upsal. Ser. 4, 1, no. 1: 76. 1905. Type: Argentina, Jujuy, Yavi in fissuris rupium, ca. 3400 m, 1–2 Jan 1902, *R.E. Fries 770* (S, lectotype).

##### Note.

With all the aspect of the typical variety, but with stems, surfaces of leaves and branches of inflorescence densely covered with minute stipitate glandular hairs.

Bolivia: Tarija: Prov. Cercado, cerca Tolomosa, suelo franco arcilloso, 1980 m, plano, flores lilas, arbusto, hasta 1.5 m, comuń, 5.5.86, *E. Bastiás 4254* (LPB, US); Prov. Arce, 39.9 km S of jct. of road to Entre Rios, on road to Pascaya, elev. 2100–2200 m, isolated pocket of *Podocarpus* forest with dry open thorn-scrub (*Acacia*, *Prosopis*) below. 21°54'S, 64°41'W., corollas bright pinkish-purple, dry open hillsides, 29 April 1983, *J.C. Solomon 10266* (MO, US); Prov. Cercado, camino a alto España 2400 m, suelo limo arenoso pH 5.2 Frec. Aislado, ca. 45 cm de alto, flor lila-rosada, 9.4.1988, *F. Ehrich 527* (LPB, US).

#### Fleischmannia pratensis

Taxon classificationPlantaeAsteralesAsteraceae

(Klatt) R.M. King & H. Rob., Phytologia 19: 205. 1970

Eupatorium schiedeanum var. tomentosum Steetz in Seemann, Bot. Voy. Herald 146. 1854. Type: Panama: Chiriquí, Volcán Chiriquí, *Seemann 1138* (holotype BM, photos MO, US).
Eupatorium schiedeanum var. capitatum Steetz in Seemann, Bot. Voy. Herald 146. 1854. Type: Panama: Chiriguí, Volcán Chiriquí, *Seemann s.n.* (holotype BM, photos MO, US).
Eupatorium pratense Klatt in T. Durand & Pittier, Bull. Soc. Roy. Bot. Belgique 31: 193. 1892. Type: Costa Rica: Savanes Boruca, Dec. 1891, *Pittier 4756* (lectotype US).
Eupatorium pacacanum Klatt, Leopoldina, Bot. Beibl. 3. 1895. Type: Costa Rica: clairières du Rodea de Pacaca, 1–2 Jan, 1891, *Pittier 3324* (holotype BR, isotype GH).
Eupatorium roseum Klatt, Bull, Soc. Roy, Belgique 31: 194. 1892 [1903]. Type: Costa Rica: clairières du Rodea de Pacaca, 1–2 Jan, 1891, *Pittier 3324* (holotype BR, isotype GH).
Fleischmannia croatii R.M. King & H. Rob., Phytologia 28: 76. 1974. Type: Panama, Chiriquí, vicinity of Las Nubes, 4.3 km NW of Río Chiriquí, Viejo, W of Cerro Punta, 2200 m, *Croat 22300* (holotype MO).
Fleischmannia panamensis R.M. King & H. Rob., Phytologia 28: 80. 1974. Type: Panama, Coclé, near La Mesa, 11 Feb. 1971, *Croat 13354* (holotype MO).

##### Description.

Erect to reclining perennial herbs or subshrubs, to 1.5 m tall; stems greenish to brown or reddish, densely puberulous to tomentellous; branches spreading at 25–90° angles. Leaves opposite; petioles slender to 3 cm long; leaf blades herbaceous, rhomboid to narrowly ovate, to 5.5 cm long, to 3.5 cm wide, base broadly acute to truncate, margins usually crenate-serrate with 6–12 teeth on each side, apex short-acute to scarcely acuminate, adaxially pilose to pilosulous, abaxially sparsely to rather densely pilosulous, puberulous on veins, with glandular dots; triplinervate from petiole. Inflorescence a broad laxly corymbiform panicle with densely corymbiform branches; peduncles 1–3 mm long, puberulous. Heads 5–6 mm high; involucral bracts 18–25 in 3–4 series, gradate, mostly short-acute, scarious margins narrow to rather broad distally. Florets 15–25 in a head; corollas lavender to white, 2.5–3.5 mm long, basal tube 0.5–1.0 mm long, throat 1.5–2.0 m long, lobes ca. 0.5 mm long, with short hairs outside, anther thecae 0.8–0.9 mm long, apical appendage ca. 0.25 mm long; style branches narrowly linear. Achenes 1.2–1.7 mm long, black with black ribs; setulae on ribs and distal faces; pappus white, of 20–30 scabrous contiguous bristles ca. 1.8–2.2 mm long.

##### Specimens seen.

Bolivia, Tipuani—Valley: Hacienda Casana, Bobuach de Abhänge, 1400 mm, 31 July 1922, *O. Buchtien* 7554 (US); Bolivia: Cochabamba, Prov. Narciso Campero Leyes, 12 km al NW de Novillero a Santiago, 18°17'S, 65°16'W, 2500 m, Bosque semideciduo, disturbado de 7 m de altura, en quebrada, con *Anadenanthera*, hierba 0.30 m, flores violetas, 17 June 1995, *N. Kessler, J. Gonzales, K. Bach & S. Hohnwald 4613* (LPB, US); La Paz, Yungas, 6000 ft., 1885, *H.H. Rusby* 1608 (NY, US); La Paz, Abel Iturralde, along road between Tumupasa and San Jose de Uchupiamonas; NW of Tumupasa along slope leading up to Parque Nacional Madidi, 5.5–5.8 km above Jct to San Jose near Tumupasa, 15°45'S, 67°50'W, 830–850 m, 21 m tall, flowers lavender, 9 August 2000, *T. Croat, A.C. Acebey & T. Kroemer 84384* (MO, US); La Paz, Nor Yungas, Region von Compatá, Hacienda “El Choro”, an Wagen zw. Gebürch, 1700 m, Wird 1 m hoch, Blüten helllila aurz-Weiss, 5 July 1930, *O. Buchtien 8209* (US); La Paz, Nor Yungas, 4.5 km below Yolosa, then 10 km W on road up the Río Huarinilla, 16°12'S, 67°50'W. 1450 m, heavily disturbed moist forest, mostly secondary growth, roadside; suffrutescent, 75 cm tall, flowers pale lavender, 19–20 Oct. 1983, *J.C. Solomon 8514* (MO, US); La Paz, Nor Yungas, en rente de Santa Rosa, Valle del Río Unduavi, 1530 m, arroyo cerco del camino; arbusto 1.2 m, flor lila, 5 9 1987, *E. Vargas & R. Seidel 457* (LPB, US); La Paz, Nor Yungas, Chairo, 16°13'S, 67°52'W, 1400 m; restos de Bosque monte. ano, abierto con mucha *Inga adenophylla*; en la orilla alta de Río Huarinilla, hierba 1.8 m, flores moradas pequeñas, 19 June 1997, *St. S. Beck 23001* (LPB, US); La Paz, Sud Yungas, etwa 5 Fahrkilometer ah der Strade von Chulumani Richtung Ocobaya, ca. 1500 m, Bluten hellblau, 16 May 1988, *T. Feuerer & P. Franken 11741* (US).

This is what has been called *Eupatorium pycnocephalum* Less. by B.L. Robinson (1920). Also, *Vargas & Seidel 457* has been previously misidentified as *Fleischmannia tamboensis* and *Beck 23001* has been misdetermined as *Fleischmannia yungasensis*.

#### Fleischmannia schickendantzii

Taxon classificationPlantaeAsteralesAsteraceae

(Hieron.) R.M. King & H. Rob., Phytologia 19: 205. 1970

Eupatorium schickendantzii Hieron., Bot. Jahrb. Syst. 22: 769. 1897. Type: Argentina. Catamarca, in der Quebrada (Schlucht) und auf der Cuesta de la Muschaca, Feb 1876, *Schickendantz 259* (holotype B, destroyed, photos GH, US).

##### Description.

Erect or reclining herbs to 1.5 m tall; stems terete, striate, puberulous; internodes 5–12 cm long; branches spreading at 45–90° angles. Leaves opposite; petioles slender, 1.5–40 cm long, puberulous; leaf blades lanceolate to narrowly ovate, 4–9 cm long, 1.8–6.5 cm wide, obtuse at base, each margin with 5–11 sharp or crenate teeth, apex narrowly acute but not acuminate; surfaces sparsely pilosulous to nearly glabrous, puberulous on veins; triplinervate from petiole, main veins dark. Inflorescence lax, with elongate branches bearing groups of few heads at corymbiform cymose tips; peduncles 5–30 mm long, puberulous. Heads with involucres 4–5 mm high, 2.5–4.0 mm wide; involucral bracts 20–26 in 5–6 series, gradate, outer bracts 1.0–1.5 mm long, ovate-lanceolate, puberulous, middle bracts lanceolate, 2.5–3.0 mm long, usually acute, inner bracts linear-oblong, 4.0–4.5 mm long, glabrous. Florets 17–24 in a head; corollas lilac, ca. 3 mm long, basal tube 0.6 mm long, throat ca. 2 mm long, lobes ca. 0.4 mm long and wide, without hairs outside; anther thecae ca. 0.8 mm long; apical appendage ca. 0.25 mm long; style branches slender, scarcely broadened distally. Achenes 2.0–2.5 mm long, ribs narrowly pale, setuliferous; pappus whitish, of ca. 30 slender bristles ca. 3 mm long, not contiguous at base.

##### Specimens seen from Bolivia.

Tarija, Prov. O’Connor, 73.i km E of Tarija-Padcaya road, on road to Entre Rios (ca. 1 km below Narvaez), elev. 1700 m, moist sub-tropical forest with many Myrtaceae, *Podocarpus*, Legumes & abundant epiphytes, 21°25'S, 64°63'W, shrub, 1.5 m, forest understory, corollas very pale pink, 1–2 May 1983, *J.C. Solomon 10333* (MO, US); Tarija, Prov. Arce, 5 km S of Comunidad Guayavillas (28.3 km S of Padcaya) on road to Bermejo. Elev. 1800–1900 m, disturbed dry forest with isolated patches of *Podocarpus*, Myrtaceae in more moist places. 22°01'S, 64°39'W, corollas white, forest understory, 6 May 1983, *J.C. Solomon 10500* (MO, US); Tarija: Prov. Arce, valley of the Río Chillaguatas, below Rancho Nogalar on trail between Sidaras and Tariquia, 22°05'S, 64°25'W, elev. 1100 m, moist sub-tropical forest, corollas white, moist sand along river, young plant, 14–16 Oct, 1983, *J.C. Solomon 11254* (MO).

#### Fleischmannia soratae

Taxon classificationPlantaeAsteralesAsteraceae

(B.L. Rob.) R.M. King & H. Rob., Phytologia 19: 206. 1970

Eupatorium soratae Sch.Bip. ex B.L. Rob., Contrib. Gray Herb. 61: 541. 1920. Type: Bolivia. La Paz: Prov. Larecaja, in woods of the temperate region in the valley of Challasuya, near Sorata, alt. 2700–2800 m, 8 Apr. 1858, *Mandon 251* (holotype NY, photos GH, US).

##### Description.

Perennial herbs to 1 m tall; stems slender, flexuous, terete, glabrate, branches few. Leaves opposite; petioles flexuous, 3–8 mm long, puberulous with short purple-jointed hairs; leaf blades thin-membranaceous, 2.0–2.5 cm long, ca. 0.8 cm wide, narrowly ovate to lanceolate, base subcuneate, margins serrate with 3 or 4 blunt teeth on each side, apex scarcely acuminate, surfaces sparsely puberulous chiefly on veins; triplinervate from petiole, main veins whitish. Inflorescence a loose panicle with short-spreading mostly opposite branches, branches terminating in cymes with 5–14 heads; peduncles slender, ca. 1–3 mm long, puberulous. Heads ca. 6 mm high; involucral bracts 10–12 in 3–4 weakly gradate series, mucronate from rather obtuse tips, outer bracts short, ovate, intermediate bracts oblong, inner bracts oblong-linear. Florets 7–9 in a head; corollas apparently white, ca. 3 mm long, basal tube ca. 0.5 mm long, throat ca. 1.5 mm long, lobes ca, 0.4 mm long, slightly hispiduous outside; anther thecae ca. 1 mm long; apical appendages ca. 0.2 mm long; style branches not broadened distally. Achenes ca. 1.5 mm long, black with pale seemingly glabrous ribs; pappus white, with ca. 30 slightly non-contiguous bristles ca. 2.5 mm long.

The NY holotype has been seen, but no other specimens have been seen that seem to be the same species. This is apparently one of the few species of the genus that can be distinguished by the fewer involucral bracts and florets in the heads. The species was described as having heads with only about 10 involucral bracts and 7 florets.

#### Fleischmannia steinbachii

Taxon classificationPlantaeAsteralesAsteraceae

H. Rob. sp. nov.

urn:lsid:ipni.org:names:77151277-1

##### Type.

**Figure 6.**
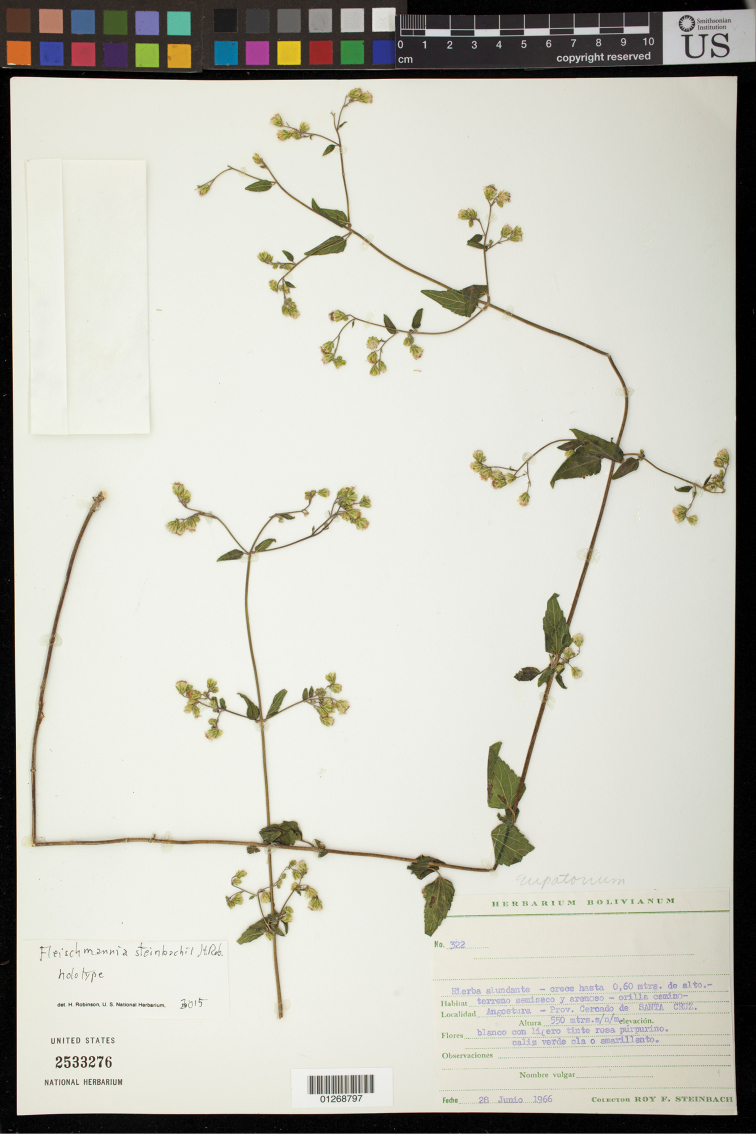
*Fleischmannia steinbachii* H.Rob., holotype (US).

Bolivia: Santa Cruz, Prov. Cercado de Santa Cruz, Angustura, alt. 550 m, hierba abundante – crecehasta 0.6 m de alto, terrano semiseco y arenoso – orilla camino, flores blanco con licero tinte rosa purpurino, 28 June 1966, *R.F. Steinbach 322* (holotype US). (Figure 6).

##### Description.

Reclining herbs to 1 m tall, stems green to pale brown, terete, striated when dry, hirsute or hirsutulous. Leaves opposite; petioles 0.7–1.5 cm long densely puberulous; blades ovate, 2–4 cm long, 1.5–2.0 cm wide, base obtuse, margins serrulate with 5–9 blunt teeth, apex narrowly acute, adaxial surface pilosulous, abaxial surface essentially concolorous, pilosulous on and between veins, triplinervate from base, with main veins prominent, usually whitish. Inflorescence, a lax panicle with many pairs of elongate mostly opposite widely divaricating branches spreading at 60–90° angles, distally with cymiform branching, each bearing 2–8 heads, main axis and main branches with foliiform bracts 1.0–2.5 cm long, 0.3–1.2 cm wide; branches and peduncles densely puberulous, peduncles 4–20 mm long. Heads ca. 6 mm high, 4 mm wide; involucre of ca. 25 strongly gradate bracts 1.0–4.5 mm long, mostly ca. 1 mm wide, few at base acute at tips, most bracts broadly rounded at tip, with narrowly scarcious margins, glabrous and with prominent pair of longitudinal veins outside. Florets ca. 17 in a head; corollas pale bluish to reddish tinged, ca. 3 mm long, basal tube ca. 0.5 mm long, throat ca. 2 mm long, lobes ca. 0.5 mm long, with few slender styliform cells on outer surface; anther thecae ca. 1 mm long; style branches not broader distally. Achenes ca. 1.8 mm long, with narrow pale, setuliferous ribs; pappus white, ca. 3 mm long, of ca. 30 slender fragile, non-contiguous bristles.

##### Paratypes.

Bolivia: Santa Cruz, Prov. Florida, 6.5 km (by road), 3 km (by air) NE of central square in Mairana on road to Yunga de Mairana, 18°06'S, 63°56'W, alt. 1800 m; near upper limit of dry woodland of *Schinopsis haenkeanaI*, with some columnar cacti (Cereus cf. dayami), beginning transition to more humid montane forest with *Clethra scabra*, *Lithtaea ternifolia*, etc., mostly turned into brushy pastures by cutting and burning, with *Baccharis* spp., *Dodonaea viscosa*, *Mimosa lepidota*. Weak, supported in shrub. Phyllaries green; flowers very pale violet, almost white, 9 May 1998, *M. Nee 49278* (NY, US); Dept. Santa Cruz, Prov. Florida: valley of Río Paredones, near Achiras Camping resort, on road to Paredones, 18°09'30"S, 63°49'W, alt. 1350–1400 m, grazed or brushy areas, dry forest and pastures; weak herb with stems 1 m long; flowers pale blue, 6 June 1998, *M. Nee 49614* (NY, US).

This new species would key in Robinson (1920) to what he called *Eupatorium tamboense* Hieron., and that is is where specimens may have been placed in the past. However, *Fleischmannia tambonsis* is described with reddish hairs and stipitate glands on the branches of the inflorescence. Some of the present paratypes have been identified in the past as *Fleischmannia polopoloensis* and *Fleischmannia schicktendanzii*. Of these the former differs by the denser more corymbiform inflorescence with white corollas. The latter differs by the more alternate distal branching of the mature plants, the more slender peduncles, the fewer and non-contiguous bristles of the pappus, and the lack of hairs or styliform papillae on the outer surfaces of the corolla lobes.

The styliform papillae on the outer surfaces of the corolla lobes have been seen in all three specimens placed in the species. The position of these papillae is one often occupied in other species by a few broader multicellular monoseriate hairs. Such styliform papillae have not been noticed in any other species in the genus, and this may prove a valuable distinguishing characteristic.

#### Fleischmannia tamboensis

Taxon classificationPlantaeAsteralesAsteraceae

(Hieron.) R.M. King & H. Rob., Phytologia 19: 206. 1970

Eupatorium tamboense Hieron., Bot. Jahrb. Syst. 22: 770. 1897. Type: Bolivia. Tarija: an der Cuesta del Tambo, between El Tambo and Narvaëz, June 1873, *Lorentz & Hieronymus 888* (holotype B, destroyed, photos GH, US).

##### Description.

Subshrub, erect, ca. 1 m tall; stems terete and glabrate below, upwardly with spreading purplish glandular pubescence, internodes to 10 cm long; branches spreading at 45–80° angles. Leaves opposite; petioles 0.5–1.5 cm long, with reddish glandular hairs; leaf blades membranaceous, rhomboid-ovate to ovate-lanceolate, to 5 cm long, to 2.2 cm wide near base, base rounded to shortly cuneate, margins each with 5–11 teeth, teeth 0.5–1.3 mm long, acute and mucronate, apex acute, adaxial surface scabridulous, abaxially scabrid on veins; triplinervaate from petiole. Inflorescence pyramidal, with slender branches ending in loose cymes; peduncles 5–6(-12) mm long, densely puberulous. Heads with involucres ca. 6 mm high, involucral bracts ca. 20–23, gradate in 5–6 series, outer ovate, ca. 1.5 mm wide, densely puberulent, greenish to purplish outside, inner bracts linear-lanceolate, to 4.5 mm long, ca. 0.8 mm wide, subacute. Florets 16–21 in a head; corollas lilac, ca. 3.2 mm long, lobes ca. 0.7 mm long and wide, short-triangular, anther thecae not seen. Achenes ca. 1.75 mm long, with ribs lighter-colored, sparsely setuliferous; pappus with ca. 20 tenuous noncontiguous bristles, ca. 3 mm long.

No specimens have been seen that match the description. The specimen that matches best is one cited by Robinson (1920) “Bolivia: La Paz, South Yungas, Sirupaya near Yanacachi, alt. 2100 m, *Buchtien 191 (300)* (NY, US)” distributed as *Eupatorium stipuliferum*. This specimen cited by Robinson has glands on the peduncles but no reddish hairs, and it is from La Paz, not Tarija. The other specimen cited by Robinson (1920) “Bolivia: Cochabamba, Río Juntas, alt. 900 m, April 1892, *Kuntze s.n.* (NY, US).” first annotated as *Eupatorium marginatum*, has densely puberulous but eglanduliferous peduncles without reddish hairs. This latter specimen remains unplaced at this time.

The species described after the Robinson 1920 treatment, *Fleischmannia yungasensis* (B.L. Rob.) R.M. King & H. Rob. seems closest to *Fleischmannia tamboensis* with its reddish hairs and stipitate glands, but it differs by its lower stems and leaves also being densely covered with stipitate glands and by its white corollas.

#### Fleischmannia yungasensis

Taxon classificationPlantaeAsteralesAsteraceae

(B.L. Rob.) R.M. King & H. Rob., Phytologia 19: 206. 1970

Eupatorium yungasense B.L. Rob., Contrib. Gray Herb. 104: 30. 1934. Type: Bolivia, Nor-Yungas, Millugnaya, alt. 1300 m, Dec 1917, *Buchtien 4407* (holotype GH; isotype US).

##### Description.

Scandent or subscandent perennial herbs, with all but most distal branches spreading at 90° angles, nearly straight or curving toward apex of main stem; stems hirtellous with reddish hairs; internodes 10–15 cm long; internodes of branchlets 2-3 cm long. Leaves opposite, leaves of main branches with petioles 10–22 mm long, densely hirtellous; leaf blades deltoid, 3.5–4.5 cm long, 1.5–2.5 cm wide, widest in basal fifth, truncate with only slight acumination at petiole; ca. 8 or 10 teeth on each margin, apex narrowly acuminate, adaxial surface hispidulous with hairs reddish near margin and pale farther from margins, abaxially spreading hirtellous on main veins, between veins sparsely pilosulous, triplinervate with strongly ascending lateral veins from basal acumination; branchlet leaves with petioles 3–5 mm long, blades 1.5–2.0 cm long, 1.0–1.7cm wide, 5–7 teeth on each margin, apex weakly acuminate to acute, pubescence as in leaves. Inflorescence with small clusters of heads terminal on elongate main stems and branches, branches of inflorescence and peduncles densely hirtellous with reddish hairs and intermixed stipitate glands; peduncles 3–7 mm long; heads ca. 6 mm high, 3–4 mm wide; involucral bracts ca. 15, in ca. 4 series, 1.5–5.0 mm long, 1.0–1.2 mm wide, basal bracts ovate and acute, more herbaceous and densely pubescent, middle and inner bracts oblong, greenish to stramineous, with mostly 2 strong longitudinal veins, margins and tips scarious, apices obtuse. Florets ca. 15–17 in a head; corollas white. ca. 3.5 mm long, basal tube ca. 0.5 mm long, throat ca. 2.5 mm long, lobes ca. 0.5 mm long, with few hairs outside, anther thecae ca. 0.8 mm long, apical appendages ca. 0.25 mm long; style branches broadened distally to 0.3 mm wide. Achenes ca. 1.7 mm long, ribs scarcely pale, sparsely setuliferous; pappus ca. 3 mm long, of ca. 35 slender bristles not broader at base, not or scarcely contiguous.

##### Specimens seen.

Bolivia: La Paz: Sud Yungas, Yanacachi, camino haxia la Chojlla a la derecho primer desvic, al borde del camino, hierba apoyandose – 1.5 cm, flores blancas, 2100m, Matorral, secondary vegetation, 7 9 1987, *E. Vargas & R. Seidel 490* (US, LPB). Bolivia. La Paz, Sud Yungas, Yanacachi, 1 km NNE de la Choilla, ladera de un arroyo, bosque bajo, 215- m.s.n.m. Trepadore colgante, 31 Oct. 1988, *Seidel 1354* (LPB, US). La Paz: Sud Yungas, bajo de Pariguaya, 16°40'S, 67°31'W, 2000 m, bosque seco, arrina del río; subarbusto, 1 m, altos, flores blancas, 30 April 1995, *St. S. Beck 22419* (LPB, US). La Paz: Inquisivi, 10 km al N de Inquisivi por el camino a Suri, al rededores del Puente sobre el Río Kato. Bosque seco con *Prosopis*, *Acacia*, *Schinopsis* y *Pereskia*, 16°48'S, 67°11'W, 2100–2200, semi-apoyante, hasta 2 m, corollas blancas, 12 march 1899, *J.C. Solomon & M. Nee 18151* (MO, US); La Paz: Inquisivi. On the slope W of the Río Khatu between the mouth of the Río Cambillua and the Río Jokho Pampa, ca. 5 km SW of Inquisivi. Mostly semi-decidous chapparal-like scrub, 16°55'S, 57°11'W, 2500–2700 m, on bank in dry woodlands. Leaves purple, flowers white. 17 Aug 1988, *Lewis 881098* (LPB, MO, US). La Paz: Inquisivi, Prov. “Huayra Pata”—Major ruin discovery of large fortelezas, irrigation canals and mysterious foundations cover this ridge which is 2 km NE of the mouth of the Río Aguilani at Lakachaka, and 2 km S of the junction of the Río Mikhailpurhua and Río Aduada. 11 km N of Choquetanga. Area of ancient ruins which has been overgrazed, burned and generally raped into semi-barren grassland with some small shrubs. Collected bordering woodlands and mattorales. 15°39'S 67°20'W, vine-like, with opposite leaves, inflorescence without rays, white, leaves rather dry and curled when collected. Shrubby woodland, 19 Nov.1991, *M. Lewis 40569* (LPB, MO, US, Quime); La Paz: Inquisivi, comunidad Khora-Lakachaca, Cuenca del Río Miguillas, 20 km de Choquetanga, 16°30'S, 67°20'W, 1450 m, Bosque, de especies semideciduos, con varias Leguminosas-Mimosas vegetación sobre terrazas antiguas, Transecto 9, herbácea terrestre, flores blancas. 30 May 1994, *M. Salinas 3061* (LPB, US). La Paz: Inguisivi, Camillaya arriba del pueblo, 16°48'S, 67°12'W, 3400 m, restos bosque alto-montano, apoyandose, tallos pargos de 4 m, flores blancas, 29 Sept. 1997, *St. S. Beck 24350* (LPB, US).

*Beck 24350*, *Solomon & Nee 18152, & Salinas 3041* previously have been misidentified as *Fleischmannia soratae*. The specimen *E. Vargas & R. Seidel 490* is evidently the apical part of a branch, with persisting primary branch leaves and a pyramidal inflorescence. The *Lewis* collections seen are evidently cut from median segments of the stems showing the consistently widely spreading branches and lacking main axis leaves.

The species is by all indications scandent, although it is probably more of a scrambling vine. The reddish pubescence is rather distinctive but according to descriptions also occurs in *Fleischmannia tamboensis* of Bolivia and such species as *Fleischmannia cookii* (B.L. Rob.) R.M. King & H. Rob. and *Fleischmannia rhodotephra* (B.L. Rob.) R.M. King & H. Rob. in Peru. *Fleischmannia tamboensis* may be closely related, with its indument of red and glandular hairs, but that is from southernmost Bolivia in Tarija, and was described by Hieronymus (1897), one of its collectors, as suffruticose to 4 m tall, and sparsely branched, with membranaceous leaves with upper surfaces “scabriusculis” and abaxial surfaces scabrid on the nerves, and with peduncles ca. 6 mm long. This latter habit agrees with the type photograph that has been seen.

## Supplementary Material

XML Treatment for Fleischmannia dissolvens

XML Treatment for Fleischmannia hassleri

XML Treatment for Fleischmannia hatschbachii

XML Treatment for Fleischmannia laxa

XML Treatment for Fleischmannia laxicephala

XML Treatment for Fleischmannia matogrossensis

XML Treatment for Fleischmannia microstemon (Cass.) R.M. King & H. Rob. var. microstemon

XML Treatment for Fleischmannia microstemon var. paniculata

XML Treatment for Fleischmannia remotifolia

XML Treatment for Fleischmannia bridgesii

XML Treatment for Fleischmannia fiebrigii

XML Treatment for Fleischmannia microstemon

XML Treatment for Fleischmannia neei

XML Treatment for Fleischmannia polopolensis

XML Treatment for Fleischmannia prasiifolia

XML Treatment for Fleischmannia prasiifolia var. glandulifera

XML Treatment for Fleischmannia pratensis

XML Treatment for Fleischmannia schickendantzii

XML Treatment for Fleischmannia soratae

XML Treatment for Fleischmannia steinbachii

XML Treatment for Fleischmannia tamboensis

XML Treatment for Fleischmannia yungasensis
